# Prevalence and associated factors of poor sleep quality among postpartum women in North West Ethiopia: a community-based study

**DOI:** 10.1186/s12888-022-04173-x

**Published:** 2022-08-08

**Authors:** Dereje Nibret Gessesse, Nuhamin Tesfa Tsega, Mastewal Belayneh Aklil, Wubedle Zelalem Temesgan, Marta Yimam Abegaz, Tazeb Alemu Anteneh, Nebiyu Solomon Tibebu, Haymanot Nigatu Alemu, Tsion Tadesse Haile, Asmra Tesfahun Seyoum, Agumas Eskezia Tiguh, Ayenew Engida Yismaw, Muhabaw Shumye Mihret, Goshu Nenko, Kindu Yinges Wondie, Birhan Tsegaw Taye, Azmeraw Ambachew Kebede

**Affiliations:** 1 Department of Clinical Midwifery, School of Midwifery, College of Medicine and Health Sciences, University of Gondar, Gondar, Ethiopia; 2 Department of Women’s and Family Health, School of Midwifery, College of Medicine and Health Sciences, University of Gondar, Gondar, Ethiopia; 3 Department of Psychiatry, College of Medicine and Health Sciences, University of Gondar, Gondar, Ethiopia; 4 School of Nursing and Midwifery, Asrat Woldeyes Health Science Campus, Debre Berhan University, Debre Berhan, Ethiopia

**Keywords:** Ethiopia, Poor sleep quality, Postpartum women

## Abstract

**Introduction:**

Although sleep disturbance is a community problem, there is limited study in Ethiopia. Therefore, this study aimed to identify the prevalence and factors affecting postpartum poor sleep quality in women.

**Method:**

A community-based cross-sectional study was conducted from July 1st to August 30th, 2021 in Gondar city. The cluster sampling method was used to address 858 study participants. The Pittsburgh Sleep Quality Index (PSQI) 19-item self-report measure of sleep quality over the past month was used to measure maternal sleep quality during the postpartum period and a global PSQI score of 5 or more was used to indicate poor sleep quality. Binary logistic regression was used to identify variable association and 95% confidence level and adjusted Odds Ratio were used to declare association.

**Result:**

Poor sleep quality prevalence during postpartum period was 24.0% (95%CI: 21.3–26.9). factors significantly associated with poor sleep quality were family size [AOR = 1.76; 95% CI: (1.14–2.73)], unplanned pregnancy [AOR = 2.11; 95%CI: (1.17–3.80)], had a family history of mental illness [AOR = 3.70; 95%CI: (2.15–6.37)], had known medical disorders [AOR = 2.59; 95%CI: (1.51–4.43)], having intimate partner violence [AOR = 2.58; 95%CI: (1.78–3.75)], and women who can read and write and who complete secondary school [AOR = 2.60; 95% CI: (1.20–5.66)] and [AOR = 2.02; 95%CI: (1.16–3.53)] respectively. On the other hand, being housewife, merchant, and government-employed [AOR = 0.32; 95%CI: (0.14–0.73)], [AOR = 0.13; 95%CI: (0.05–0.34)], and [AOR = 0.38; 95%CI: (0.14–0.98)] respectively were identified to be factors significantly associated with poor sleep quality.

**Conclusion:**

Poor sleep quality prevalence is high in the community of Gondar city. Thus, setting strategies to increase women’s educational level, providing health education programs to create awareness on the consequence of intimate partner violence which could reduce the violence; increasing screening for medical disorders before or during maternity period, preventing unplanned pregnancy with effective family planning method, and employing women in a certain organization will have a great role in reducing poor sleep quality.

## Introduction

Sleep is a physiological process and plays a vital role in the physical, emotional, and mental health of human beings [1, 2]. Sleep disorders involve problems with the quality, timing, duration, and amount of sleep. The third edition of the international classification of sleeping disorders identified seven major categories of sleeping disorders such as insomnia disorders,sleep-related breathing disorders, central disorders of hypersomnolence, circadian rhythm sleep-wake disorders, sleep-related movement disorders, parasomnias, and other sleep disorders [3]. Sleep quality was a difficult term to clearly define because its concept varies from one person to another as subjective expectancy is highly variable and complex [4]. Both subjective and objective approaches can be used to assess an individual’s subjective perception of his or her sleep. The subjective method, Pittsburgh Sleep Quality Index (PSQI) is a widely used questionnaire to measure sleep quality [5]. In women, pregnancy and the postnatal period may be more susceptible to the development of Insomnia and other sleep problems [6]. Postpartum physiological, psychological, and social changes have an impact on sleep quality [7, 8]. In addition, women propose various reasons for poor sleep quality during a postpartum period including postpartum physical discomfort, changes in home responsibility, financial stressors, and insufficient time due to increased caregiving demands [9–11].

Globally, it is estimated that 30 to 40% of the general population is affected by different types of sleep disorders. A meta-analysis conducted on pregnant and postpartum women revealed that 44.5% of pregnant and 67.2% of postpartum women experience poor sleep quality [5]. Sleep issues are one of the first signs of the most common mental illnesses, such as depression, anxiety, and alcoholism [1]. Sleep problems during or before the postpartum period are a severe maternal health problem that happens at a critical moment in a mother’s life and leads to a variety of negative consequences for partners as well as emotional, behavioral, and sleep problems in infants [12]. Poor postpartum sleep quality has been correlated to employed women, having male newborns, home birth, mental illness during pregnancy, and medical issues of the mother or baby after delivery [13].

Postpartum sleep quality is an important indicator of women’s physical and mental health, but there is no study in Ethiopia, so this study aims to assess the prevalence and factors associated with poor sleep quality among women in the postpartum period.

## Methods and materials

### Study design, period, and area

A community-based cross-sectional study was conducted in Gondar city from July 1st to August 30th, 2021. The city is found in Amhara national regional state, Central Gondar Zone. It is located 750 km Northwest far from Addis Ababa which is the capital city of Ethiopia. There are 1 governmental referral hospital, 8 governmental health centers, 22 health posts, 1 private primary hospital, and 1 general hospital serving the community in Gondar city.

### Study population and eligibility criteria

All women who gave birth in the last 12 months in selected clusters of Gondar city during the data collection period and residing for at least 6 months before the data collection period were included in the study. However, women who were critically ill and unable to give a response throughout the data collection period were excluded.

### Sample size determination and sampling procedure

The sample size for the current study was determined using the assumptions of a single population formula considering the following assumptions: the proportion of poor sleep quality-50%, level of confidence-95%, and margin of error-5%. Therefore, the sample size (n) $=\frac{{\left(\mathrm{Z}\upalpha /2\right)}^2\mathrm{p}\left(1-\mathrm{p}\right)}{{\mathrm{d}}^2}\frac{={(1.96)}^{2\ast }0.5\left(1-0.5\right)}{(0.05)2}$ = 384. After considering a design effect of 2 and a non-response rate of 10%, we obtained a total sample size of 845. There are 22 kebeles in Gondar city. From which seven kebeles (30% of the total kebeles) were selected by the simple random sampling using lottery method. A house-to-house visit was carried out in the selected kebeles (clusters) to find eligible women for the study, households had enumerated and the identified households had revisited. All eligible women in the selected clusters were interviewed after written informed consent had taken using structured questionnaire through face-to-face interviews . Finally, due to the nature of cluster sampling, 858 women were interviewed. Revisist was taken to address a women missed in the first visit. There was no none response while we collect a data during our data collection period.

### Variables of the study

Maternal sleep quality was the outcome variable, whereas maternal age, maternal occupation, monthly income, religion, mother’s educational status, marital status, family size and exposure to media, parity, having antenatal care (ANC) visits, the number of ANC visits, postnatal care (PNC) visit, Number of PNC visit, visited by Health Extension Workers, Place of the recent delivery, assistant of the delivery, history of neonatal death, distance to the health facility, women’s autonomy, pregnancy planeness, intimate partner violence, husband educational status, husband occupation, and husband involvement in Maternal, Neonatal and Child Health.

### Operational definitions and measurements

#### Sleep quality

The Pittsburgh Sleep Quality Index (PSQI) 19-item self-report measure of sleep quality over the past month was used to measure maternal sleep quality during the postpartum period. The tool used to measure outcome variable had a diagnostic sensitivity of 82% and specificity of 56.2% at greater than five cutoff value [14]. PSQI consists of 7 component scores (ranging from 0 to 3), measuring subjective sleep quality, sleep latency, sleep duration, sleep efficiency, sleep disturbances, use of sleeping medication, and daytime dysfunction. The 7 component scores are summed to give a global PSQI score ranging from 0 to 21, with higher scores reflecting greater overall sleep disturbances. A global PSQI score of 5 or more indicates poor sleep quality [11].

#### Husband/partner involved in MNCH services

Based on the summative score of variables designed to assess husband involvement with a score equal to or above the mean was considered as involved [15].

#### Household decision-making power

The ability of women to act independently on the household activities including their health, children’s health, freedom of movement, and control over finance without asking permission from another person. Thus, based on the summative score of variables designed to assess household decision-making power women who were answered above the median value were considered as having higher decision-making power [16].

#### Intimate partner violence

Intimate partner is considered as a current spouse, co-habited, current boyfriend, former partner, or spouse. The woman was considered to have experienced intimate partner violence if she reported experiencing any one of the ranges of sexual, psychological, and physical or any combination of the three coercive acts regardless of the legal status of the relationship with her current/former intimate partner [17].

#### Social support

The Oslo Social Support Scale (OSS-3) scores ranged from 3 to 14 with a score of 3–8, poor support; 9–11, moderate support; and 12–14, strong support [18].

### Data collection tools, methods, and procedures

The data collection tool was developed by reviewing the literature [5, 8, 11, 13, 19–22] and was collected using a structured questionnaire through face-to-face interviews. The questionnaire contains socio-demographic characteristics, obstetric and maternal health services-related characteristics, decision-making autonomy, intimate partner violence-related questions, social support, husband involvement, and questions assessing maternal sleep quality. Seven Bachlor Science and three Master Science in Midwifery were trained in the interview technique, collected and supervise the data process, respectively.

### Data quality control

Initially, the questionnaire was prepared in English and translated to the local language, and back to English to check the consistency. Before the actual data collection, a pretest was done on 5% of the sample size out of the study sites to check its understandability, logical sequence, and ease for respondents. The one-day training was given to data collectors and supervisors. During data collection, the questionnaire was checked for completeness daily by the supervisors.

### Data processing and analysis

Data was intered to EPI DATA version 4.6.0 software and data cleaning and analysis was done using Package for the Social Sciences (SPSS) version 20 software. Descriptive statistics were done for variables; socio-demographic characteristics, reproductive and maternal health service characteristics of participants. Variables that fulfilled the chi-square assumption were included in bivariable logistic regression to identify independent factors and variables having a *p*-value of less than 0.2 were included in the multivariable logistic regression analysis to control possible confounders. In the multivariable logistic regression analysis, a *p*-value of ≤0.05 with 95% CI for the odds ratio was used to determine the level of significance.

### Ethical considarations

The study was conducted under the Ethiopian Health Research Ethics Guideline and the declaration of Helsinki. The study involved human study participant. Ethical clearance was obtained from the institutional review board of the University of Gondar. A formal support letter was obtained from the selected clusters (kebeles) administrators of Gondar city. The study participants were aware as they have the right to decline to participate anytime during interview. After a clear explanation of the aim of the study, written informed consent was taken from each study participant.

## Results

### Socio-demographic characteristics of study participants

In the current study, about 858 participants were enrolled making the response rate 100%. The mean age of study participants was 29.53(±4.79 standard divation) and about 43.2% were between 24 to 29 years of age. About 381(44.4%) participants’ occupations were housewives and 371(43.2%) participants’ level of education was diploma and above. Most (82.3%) of the study participants were orthodox Christian religion followers. More than half (56.9%) of study participants’ family size was less than five. About 82.3% of participants had media exposure and only 10% had medical problems as shown in Table 1.

**Table 1:** Socio-demographic characteristics of the study participants in Gondar city, northwest Ethiopia, 2021 (*n* = 858)

Variables	Frequency	Percentage (%)
**Age of women**		
18–23	74	8.6
24–29	371	43.2
30–35	319	37.2
≥ 36	94	11.0
**Women’s educational level**		
Can’t read and write	50	5.9
Can read and write	55	6.4
Primary education	140	16.3
Secondary education	242	28.2
Diploma and above	371	43.2
**Women’s occupation**		
House wife	381	44.4
Merchant	105	12.2
Government employed	239	27.9
Private employed	99	11.5
Student	34	4.0
**Religion**		
Orthodox	706	82.3
Muslim	107	12.5
Others ^a^	45	5.2
**Marital status**		
Married	779	90.8
Unmarried	79	9.2
**Family size**		
1–4	488	56.9
≥ 5	370	43.1
**Media exposure**		
Yes	706	82.3
No	152	17.7
**Have medical problem**		
Yes	86	10.0
No	772	90.0

^a^Protestant and Catholic

### Reproductive and maternity health service characteristics of the study participants

Regarding reproductive and maternity health care service, most (86.8%) pregnancy was planned. About 794 (92.5%) of last pregnancy were supported by their families. About 97.3% of study participants had antenatal care follow up from which 61.7% had more than 4 antenatal care visits. More than two-thirds (70.2%) of study participants had given birth to three or more. Around half (48.6%) of mothers had intimate partner violence during their last pregnancy as shown in Table 2.

**Table 2:** Reproductive and maternity health service characteristics of the study participants in Gondar city, northwest Ethiopia, 2021 (*n* = 858)

Variables	Frequency	Percent (%)
**ANC follow up**		
Yes	835	97.3
No	23	2.7
**Number of antenatal care visits (*n*** **= 835)**		
1–3	320	38.3
≥ 4	515	61.7
**Parity**		
1–2	602	70.2
≥ 3	256	29.8
**Last pregnancy planned**		
Yes	745	86.8
No	113	13.2
**Intimate partner violence**		
Yes	417	48.6
No	441	51.4
**Pregnancy supported by family**		
Yes	794	92.5
No	64	7.5

*ANC* Antenatal Care

### Prevalence and associated factors of poor sleep quality

In this study, about 24.0% of women have experienced poor sleep quality during their most recent postpartum period (95%CI: 21.3–26.9).

In the multivariable logistic regression, variables such as family size, maternal educational status, unplanned pregnancy, having a medical disorder, history of mental illness in a family, maternal occupation, and intimate partner violence were factors significantly associated with poor sleep quality during the postpartum period.

Thus, the odds of poor sleep quality were 1.8 times more likely among women who had a family size of less than five compared to women whose family size is greater than four [AOR = 1.76; 95% CI: (1.14–2.73)]. The odds of poor sleep quality were also 2.6 and 2.0 times higher among women who can read and write and who complete secondary school compared to college and above [AOR = 2.60; 95% CI: (1.20–5.66)] and [AOR = 2.02; 95%CI: (1.16–3.53)] respectively. Likewise, the odds of poor sleep quality were 2.1 times higher among mothers who had planned pregnancy than their counterparts [AOR = 2.11; 95%CI: (1.17–3.80)]. Moreover, mothers who had known medical disorders during recent pregnancy were 2.6 times more likely to experience poor sleep quality than healthy mothers [AOR = 2.59; 95%CI: (1.51–4.43)]. Similarly, the odds of poor sleep quality were 3.7 times more likely among women who had a family history of mental illness compared to their counterparts [AOR = 3.70; 95%CI: (2.15–6.37)]. This study also showed that the odds of poor sleep quality were 2.6 times higher among women who had experienced intimate partner violence compared to their counterparts [AOR = 2.58; 95%CI: (1.78–3.75)]. Finally, women whose occupation was housewife, merchant, and government-employed were 68, 87 and 62% less likely to experience poor sleep quality compared to women whose occupation was student respectively [AOR = 0.32; 95%CI: (0.14–0.73)], [AOR = 0.13; 95%CI: (0.05–0.34)], and [AOR = 0.38; 95%CI: (0.14–0.98)] (Table 3).

**Table 3:** Bivariable and multivariable logistic regression analysis of associated factors of poor sleep quality in Gondar city, Northwest Ethiopia, 2021

Variables	Sleep Quality		COR(95%CI)	AOR(95%CI)
	Good	Poor		
**Age**				
18–23	44	30	2.23 (1.15–4.34)	1.32 (0.60–2.93)
24–29	281	90	1.05 (0.62–1.79)	0.69 (0.36–1.32)
30–35	255	64	0.82 (0.47–1.42)	0.66 (0.35–1.22)
36 and above	72	22	1	1
**Family size**				
1–4	350	138	1.75 (1.26–2.43)	**1.76 (1.14–2.73)**
5 and above	302	68	1	1
**Mass media exposure**				
No	529	177	1.42 (0.915–2.20)	1.35 (0.82–2.22)
Yes	123	29	1	
**Maternal education**				
Cannot read and write	31	19	3.00 (1.59–5.64)	2.15((0.95–4.86)
Can read and write	32	23	3.51 (1.93–6.41)	**2.60 (1.20–5.66)**
Primary	111	29	1.28 (0.78–2.09)	1.30 (0.67–2.51)
Secondary	170	242	2.07 (1.41–3.05)	**2.02 (1.16–3.53)**
College and above	308	63	1	1
**Was pregnancy planned**				
Yes	587	158	1	1
No	65	48	2.74 (1.82–4.14)	**2.11 (1.17–3.80)**
**Pregnancy supported by family**				
Yes	611	183	1	1
No	41	23	1.87 (1.10–3.20)	0.65 (0.30–1.40)
**Having medical disorder**				
Yes	48	38	2.85 (1.80–4.50)	**2.59 (1.51–4.43)**
No	604	168	1	1
**History of Mental illness in a family**				
Yes	35	44	4.79 (2.97–7.71)	**3.70 (2.15–6.37)**
No	617	162	1	1
**Religion**				
Orthodox Christian	550	156	1	1
Muslim	73	34	1.64 (1.05–2.56)	1.38 (0.82–2.32)
Others^a^	29	16	1.95 (1.03–3.67)	1.59 (0.76–3.33)
**Maternal occupation**				
Housewife	283	98	0.31 (0.15–0.63)	**0.32 (0.14–0.73)**
Merchant	87	18	0.18 (0.08–0.43)	**0.13 (0.05–0.34)**
Government employee	199	40	0.18 (0.08–0.38)	**0.38 (0.14–0.98)**
Self employed	67	32	0.43 (0.19–0.94)	0.45 (0.18–1.13)
Student	16	18	1	1
**Having intimate partner violence**				
No	377	64	1	1
Yes	275	142	3.04 (2.18–4.25)	**2.58 (1.78–3.75)**

^a^protestant and catholic

## Discussion

In this study, about 24.0% of women have experienced poor sleep quality during their most recent pregnancy (95%CI: 21.3–26.9). The current study finding is lower than studies done elsewhere (28.2–96.2%) [5, 7, 8, 13, 23, 24]. This variation might be due to socio-cultural differences, all of which are done outside Ethiopia. The other reason for disparity might be due to the time of data collection and most studies were collected within 7 weeks of the postpartum period. Study result inconsistency is also due to the source of data, the tool used, and the sampling method they went through. For instance, this finding is lower than a study done in Korea (96.2%) [25]. The study in Korea was institution-based from which women may seek care for different problems including sleep disturbance which may increase the result. The other possible explanation might be the study was done among women within 6 weeks of the postpartum period from which most women suffer from a sleep problem before adaptation. This finding is also lower than the study conducted elsewhere (90%) [24]. Unlike our study, this study used a small sample, and with a different tool which may increase the prevalence. The current study is also lower than the comparative study in Taiwan (41.25 and 66.2%) [9]. The study done in Taiwan was institution-based from which women may seek care for different problems including sleep disturbance that may increase the result. Variation is also the data collection tool used (Postpartum Physical Symptoms Checklist, period of data collection (12–24 week postpartum period), and method of interview (telephone).

The odds of poor sleep quality were 1.8 times more likely among women who had a family size of less than five compared to women whose family size is greater than four. This finding is supported by another study [7, 26]. This may be explained by a woman who had a large family size could have adaptation on how to support her family and raring her baby which may increase sleep quality. Another possible explanation could be women having large family sizes might discuss the issue and made solutions for the existing problem and this might improve women’s sleep quality.

This study also showed that the odds of poor sleep quality were 2.6 times higher among women who had experienced intimate partner violence compared to their counterparts. This could be explained by intimate partner violence could end up in relationship dissatisfaction. Relationship dissatisfaction results in poor sleep quality and this was supported by evidence done in Taiwan [27].

Likewise, women having known medical disorders during recent pregnancy were 2.6 times more likely to experience poor sleep quality than healthy women. It is possible that women with physical symptoms may suffer from sleep problems, especially if left untreated. Women facing such problems most of the time think about the negative consequences of their life and this might have an impact on sleeping quality.

The odds of poor sleep quality were also 2.6 and 2.0 times higher among women who were able to read and write and have secondary education compared to women having higher education respectively. This result is supported by another study [28]. Women having higher educational level might know the coping mechanism to get good sleep compared to their counterparts.

The odds of poor sleep quality was 2.1 times higher among mothers who had unplanned pregnancy than their counterparts. The possible explanation might be women with planned pregnancies may not worry about support and child-rearing compared to women with unplanned pregnancies.

Similarly, the odds of poor sleep quality were 3.7 times more likely among women who had a family history of mental illness compared to their counterparts. It is a fact that mental illness increases in the offspring of parents with a history of mental illness [29] and this might increase the risk of sleep disorder in postpartum women. Finally, women whose occupation is housewife, merchant, and government-employed were 68, 87, and 62% less likely to experience poor sleep quality compared to women whose occupation was a student. Women whose occupation is students might fear for dropout of school which may result worry and in poor quality sleep and this is in line with studies done in Iran [29].

### Limitation of the study

In this study, PSQI tool was used which is only validated on adults in Ethiopia, but the validity was done with sensitivity of 82%, specificity of 56.2% and the specificity is low [14].

## Conclusion

Poor sleep quality prevalence among postpartum women is found to be high in the community of Gondar city. In the current study, having a family size of less than four or less, experiencing intimate partner violence, having a family history of mental illness, having a medical illness, women who were able to read and write and secondary school, having unplanned pregnancy, whose occupation is housewives, merchants, and government-employed were factors significantly associated with poor sleep quality. Thus, setting strategies to increase women’s educational level, providing health education programs to create awareness on the consequence of intimate partner violence wich could reduce the violence; increasing screening for medical disorders before or during maternity period, preventing unplanned pregnancy with effective family planning method, and employing women in a certain organization will have a great role in reducing poor sleep quality.

## Data Availability

The dataset is available from the corresponding author on reasonable request.

## Abbreviations

ANC: Antenatal care
PNC: Postnatal care
PSQI: Pittsburgh Sleep Quality Index
AOR: Adjusted Odds Ratio
COR: Crude Odds Ratio
